# 

**Published:** 1871-12

**Authors:** 


### CONTENTS.

COMMUNICATIONS

Chemical Nature of Malaria ..... 501
Anæsthesia ..... 511
How to Spell and Pronounce Aluminium ..... 514
Are we Progressing? ..... 514

PROCEEDINGS OF SOCIETIES.

Second Annual Meeting of the New Jersey State Dental Society ..... 516
First District Dental Society of the State of New York ..... 521
Proceedings of the Brooklyn Dental Society ..... 528
East Tennessee Dental Association ..... 529
Proceedings of the Tennessee Dental Society ..... 530
Eastern Indiana Dental Society ..... 531

SELECTIONS.

Dentigerous Cysts ..... 532
Disease of the Antrum ..... 540

EDITORIAL

The Ohio State Dental Society ..... 542
Change Editorial ..... 543
Close of the Volume ..... 543
Kentucky State Dental Society ..... 544

